# Time-dependent changes in the microenvironment of injured spinal cord affects the therapeutic potential of neural stem cell transplantation for spinal cord injury

**DOI:** 10.1186/1756-6606-6-3

**Published:** 2013-01-08

**Authors:** Soraya Nishimura, Akimasa Yasuda, Hiroki Iwai, Morito Takano, Yoshiomi Kobayashi, Satoshi Nori, Osahiko Tsuji, Kanehiro Fujiyoshi, Hayao Ebise, Yoshiaki Toyama, Hideyuki Okano, Masaya Nakamura

**Affiliations:** 1 Department of Orthopaedic Surgery, 35 Shinanomachi, Shinjuku, Tokyo, 160-8582, Japan; 2 Department of Physiology, Keio University School of Medicine, 35 Shinanomachi, Shinjuku, Tokyo, 160-8582, Japan; 3 Department of Orthopaedic Surgery, Saitama Social Insurance Hospital, 4-9-3 Kitaurawa, Urawa, Saitama, 330-0074, Japan; 4 Department of Orthopaedic Surgery, Murayama National Hospital Organization Murayama Medical Center, 2-37-1 Gakuen, Musashimurayama, Tokyo, 208-0011, Japan; 5 Genomic Science Laboratories, Dainippon Sumitomo Pharma Co, Ltd., 2-6-8 Doshoumachi, Chuo, Osaka, 541-0045, Japan

**Keywords:** Spinal cord injury, Neural stem/progenitor cells, Cell transplantation, Chronic phase, Microenvironment

## Abstract

**Background:**

The transplantation of neural stem/progenitor cells (NS/PCs) at the sub-acute phase of spinal cord injury, but not at the chronic phase, can promote functional recovery. However, the reasons for this difference and whether it involves the survival and/or fate of grafted cells under these two conditions remain unclear. To address this question, NS/PC transplantation was performed after contusive spinal cord injury in adult mice at the sub-acute and chronic phases.

**Results:**

Quantitative analyses using bio-imaging, which can noninvasively detect surviving grafted cells in living animals, revealed no significant difference in the survival rate of grafted cells between the sub-acute and chronic transplantation groups. Additionally, immunohistology revealed no significant difference in the differentiation phenotypes of grafted cells between the two groups. Microarray analysis revealed no significant differences in the expression of genes encoding inflammatory cytokines or growth factors, which affect the survival and/or fate of grafted cells, in the injured spinal cord between the sub-acute and chronic phases. By contrast, the distribution of chronically grafted NS/PCs was restricted compared to NS/PCs grafted at the sub-acute phase because a more prominent glial scar located around the lesion epicenter enclosed the grafted cells. Furthermore, microarray and histological analysis revealed that the infiltration of macrophages, especially M2 macrophages, which have anti-inflammatory role, was significantly higher at the sub-acute phase than the chronic phase. Ultimately, NS/PCs that were transplanted in the sub-acute phase, but not the chronic phase, promoted functional recovery compared with the vehicle control group.

**Conclusions:**

The extent of glial scar formation and the characteristics of inflammation is the most remarkable difference in the injured spinal cord microenvironment between the sub-acute and chronic phases. To achieve functional recovery by NS/PC transplantation in cases at the chronic phase, modification of the microenvironment of the injured spinal cord focusing on glial scar formation and inflammatory phenotype should be considered.

## Background

The injured spinal cord exhibits little spontaneous recovery and, as a result, many spinal cord injury (SCI) patients suffer from permanent functional impairments, such as motor and sensory dysfunction, and bladder and rectal disturbance. However, some previous reports have shown that neural stem/progenitor cells (NS/PCs) transplanted into the injured spinal cord of rodents [1-6] and non-human primates [7], 7–10 days post-injury (DPI), promote functional recovery after SCI. These reports indicate that NS/PC transplantation has therapeutic potential for SCI when performed during the sub-acute phase. However, patients continue to seek new therapies for SCI many years after their original injury, and most are therefore in the chronic phase. Although many researchers have sought to achieve functional recovery at the chronic phase of SCI by NS/PC transplantation, with one exception [8], no significant recovery of motor function has been obtained in animal models of chronic-phase SCI [9-12]. Despite differences in the survival rate, the cell types derived from the grafted NS/PCs and the distribution of grafted cells transplanted at the sub-acute versus the chronic phase remain unknown. Thus, it remains unanswered as to why grafted NS/PCs do not exert therapeutic benefits in the injured spinal cord at the chronic phase. To address this question, this study analyzed fetus-derived NS/PCs transplanted into the injured spinal cord of mice at 9 DPI and 42 DPI.

To assess the survival rate of grafted cells, we performed quantitative analysis using bioluminescence imaging (BLI) on a weekly basis until 42 days after transplantation. BLI is a powerful tool for the detection of exclusively living grafted cells that stably express luciferase in living animals after administration of luciferin, the luciferase substrate, because the luciferin-luciferase reaction depends on oxygen and ATP [13]. In this study, no significant difference in the survival rate of grafted cells between the sub-acute and chronic transplanted (TP) groups was observed at each experimental time point. Immunohistology also revealed no significant difference in the differentiation pattern of grafted NS/PCs between the two groups. In addition, inflammatory cytokines and growth factors, which influence the survival rate and differentiation characteristics of grafted cells, were expressed at similar levels at both phases. By contrast, the grafted cells were distributed broadly from the epicenter to rostral and caudal sites in the sub-acute TP group, whereas they remained near the lesion epicenter, due to extensive glial scarring, in the chronic TP group. Moreover, prominent macrophages distributed at and around the lesion epicenter in the sub-acute phase by immunohistochemistry, and microarray analysis demonstrated that the expression of Arginase-1, which is associated with M2 macrophages, was up-regulated significantly at the sub-acute phase than the chronic phase. These findings indicated that the characteristics of post-SCI inflammation are different between the sub-acute and chronic phases.

Consequently, the grafted NS/PCs did not promote any motor functional or histological recovery in the chronic TP group, while the sub-acute TP group demonstrated significant recovery compared with the vehicle control group. Taken together, these data suggest that a combination therapy of NS/PC transplantation with control of glial scar formation or inflammatory reaction may be critical to achieving functional recovery for chronic SCI.

## Results

### *In vitro* characterization of transgenic mouse-derived NS/PCs that ubiquitously express fluorescent protein-fused luciferase (ffLuc)-cp156

**Figure 1:**
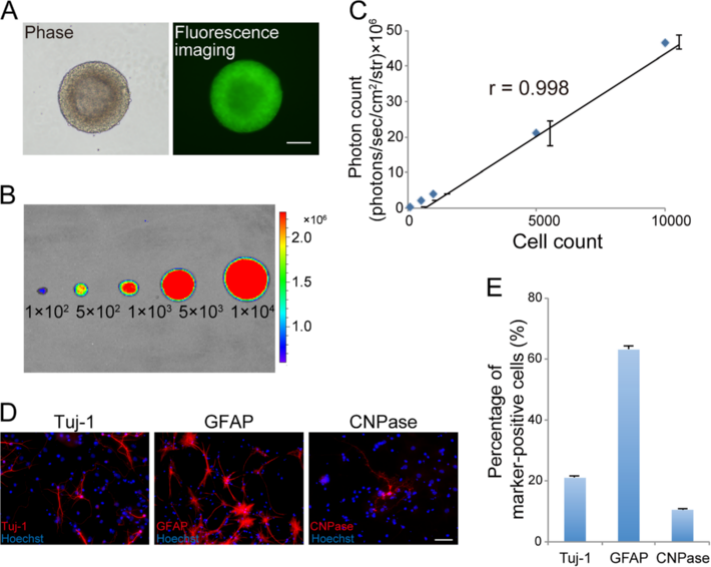
***In vitro *characterization of the NS/PCs derived from transgenic mice that ubiquitously express ffLuc-cp156. A**, Fluorescence image showing NS/PCs derived from fetal transgenic mice expressing the fluorescent protein cp156-Venus, which is originally modified from GFP. **B**, Bioluminescent signals originating from the luciferase were detected in the NS/PCs by BLI. **C**, The number of NS/PCs was significantly correlated with the measured photon count by BLI. Values are means ± SEM (n = 3). **D**, Tertiary NS/PCs differentiated into Tuj-1^+^ neurons, GFAP^+^ astrocytes, and CNPase^+^ oligodendrocytes *in vitro*. **E**, Quantitative analysis of Tuj-1^+^ neurons, GFAP^+^ astrocytes, and CNPase^+^ oligodendrocytes *in vitro*. Values are means ± SEM (n = 5). Scale bar: 100 μm in (A) and 50 μm in (D).

To identify and monitor the grafted cells by bio-imaging, a transgenic mouse that ubiquitously expresses ffLuc-cp156 was previously developed [14]. NS/PCs derived from this transgenic mouse showed strong and stable emission of ffLuc-cp156 *in vitro* (Figure 1A, B). The number of NS/PCs and the photon counts measured by BLI were significantly correlated (Figure 1B, C).

We proceeded to perform differentiation and proliferation assays of these NS/PCs *in vitro*. The NS/PCs differentiated into βIII tubulin (Tuj-1)^+^ neurons (21.0 ± 0.5%), glial fibrillary acidic protein (GFAP)^+^ astrocytes (63.0 ± 1.5%), and 2’3’-cyclic nucleotide 3’-phosphodiesterase (CNPase)^+^ oligodendrocytes (10.4 ± 0.5%) *in vitro* (Figure 1D, E). ATP production, an indirect measurement, was used to assess NS/PC proliferative ability [15]. The doubling time of the NS/PCs was 28.8 ± 0.8 h. The differentiation rate and proliferative ability of NS/PCs obtained from the transgenic mice were equivalent to those previously reported for wild-type NS/PCs [16,17].

### Comparison of the injured spinal cord microenvironment between the sub-acute and chronic phases

**Figure 2:**
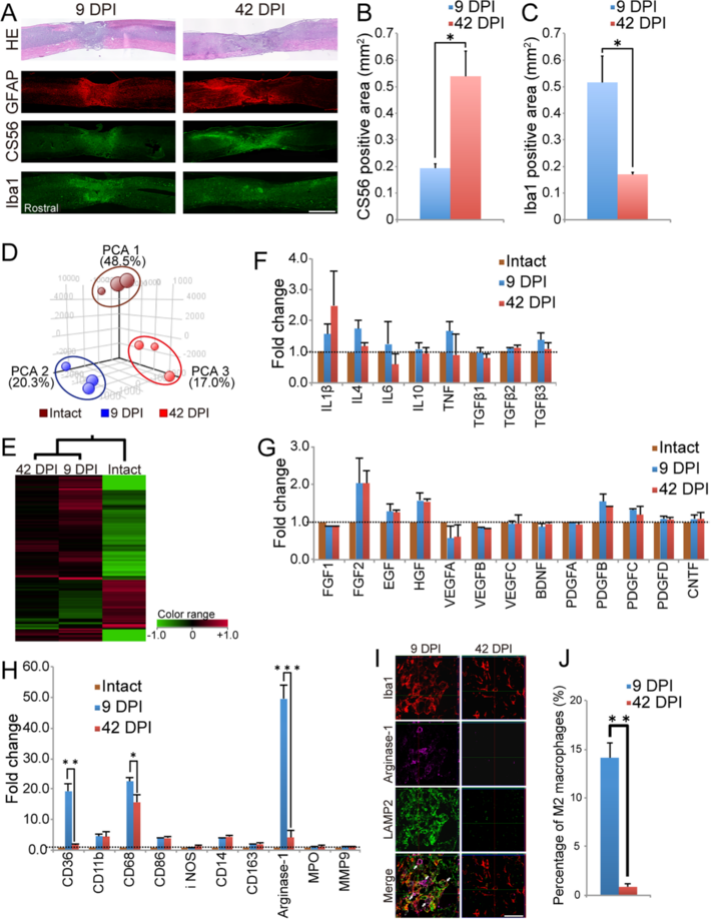
**Comparison of the microenvironment of the injured spinal cord at 9 DPI versus 42 DPI. A**, Representative images of HE staining and immunofluorescence staining for GFAP, CS56, and Iba1 in sagittal sections. **B**, CSPGs accumulation was more prominent at the lesion site at 42 DPI than at 9 DPI. Values are means ± SEM (n = 3). ^*^P < 0.05. **C**, More Iba1-positive cells were distributed at the lesion site at 9 DPI than at 42 DPI. Values are means ± SEM (n = 3). ^*^P < 0.05. **D**, Overview of all the microarray data by PCA. The samples of each group were clustered at different locations on 3D visualization. **E**, Hierarchical clustering analysis showed that the gene expression pattern at 9 DPI was similar to that at 42 DPI. However, the magnitude of changes in gene expression differed between the two injury groups. Green tiles show downregulated genes and red tiles indicate upregulated genes. **F-H**, Gene expression signals of cytokines (**F**), growth factors (**G**), and markers of inflammatory cells (**H**) at 9 DPI and 42 DPI. The gene expression levels of markers associated with microglia/macrophages significantly differed between 9 DPI and 42 DPI, but those of all cytokines and growth factors did not. Data are the mean fold-change values versus intact samples. Values are means ± SEM (n = 3). ^*^P < 0.05, ^**^P < 0.01, ^***^P < 0.001. **I**, Representative images of immunofluorescence staining for Iba1, arginase-1, and LAMP2 in sagittal sections of the lesion epicenter at 9 DPI and 42 DPI. Arrows: Iba1^+^/arginase-1^+^/LAMP2^+^ triple-positive cells. **J**, At the lesion epicenter, more Iba1^+^/arginase-1^+^ M2 macrophages had infiltrated at 9 DPI compared with 42 DPI. Values are means ± SEM (n = 3). ^**^P < 0.01. Scale bar: 1000 μm in (**A**) and 50 μm in (**J**).

To clarify differences in the microenvironment of the injured spinal cord between the sub-acute and chronic phases, histological analyses of spinal cord tissues at 9 DPI and 42 DPI were performed. Spinal cord atrophy and glial scar formation were more prominent at and around the lesion epicenter at 42 DPI than at 9 DPI (Figure 2A). A significantly larger CS56^+^ chondroitin sulfate proteoglycan (CSPG) area was detected at the lesion site at 42 DPI than at 9 DPI (Figure 2A, B). Furthermore, Iba1^+^ macrophages infiltrated area was more prominent at the lesion site at 9 DPI but not at 42 DPI (Figure 2A, C).

To analyze the gene expression profile in the injured spinal cord, we performed microarray analysis to provide a global analysis of the gene expression profile of spinal cord tissues at 9 DPI and 42 DPI. As a control, samples of uninjured naïve spinal cord were prepared. Principal component analysis (PCA) of all the microarray data revealed that the samples of the intact, 9 DPI and 42 DPI groups were clustered at different locations (Figure 2D). Hierarchical clustering of the target genes, which were narrowed down by cut-off values for expression levels and by fold change, revealed that the gene expression profiles of both injured groups were dramatically different from that of the intact group. Furthermore, the gene expression pattern at 9 DPI was similar to that at 42 DPI, but the magnitude of changes in gene expression differed between the two injury groups. At 9 DPI, the magnitude of gene expression changed more remarkably than that at 42 DPI (Figure 2E).

Subsequently, we focused on individual gene expression levels. No significant differences were observed in the expression levels of genes for individual inflammatory cytokines or growth factors between the two injury groups (Figure 2F, G). By contrast, the expression of CD36 and CD68, which are expressed on monocytes and macrophages, was significantly elevated at 9 DPI (Figure 2H). These results were consistent with the immunostaining results for Iba1 (Figure 2A, C). Interestingly, the expression of arginase-1, which is associated with anti-inflammatory M2 macrophages, was also significantly higher at 9 DPI than at 42 DPI, whereas no significant differences were observed in the expression levels of genes associated with pro-inflammatory M1 macrophages, such as CD86 or inducible nitric oxide synthase (iNOS). Consistent with these findings, immunohistological analysis revealed more Iba1 and arginase-1 double-positive cells at 9 DPI (14.1 ± 1.5%) than at 42 DPI (0.9 ± 0.4%) (Figure 2I, J).

We also evaluated the phagocytic activity of the macrophages recruited into the injured spinal cord by performing immunohistochemistry for LAMP2, a marker for endosomes or lysosomes. At 9 DPI, substantial numbers of infiltrating Iba1, arginase-1, and LAMP2 triple-positive cells were observed at and near the lesion epicenter. In contrast, only a small amount of LAMP2-positive phagocytes were localized to the lesion epicenter at 42 DPI (Figure 2I). In addition, no significant expression of myeloperoxidase, a marker of neutrophils, was observed at either 9 DPI or 42 DPI (Figure 2H).

### Comparison of survival rates and differentiation phenotypes of the grafted NS/PCs between the sub-acute and chronic TP groups

**Figure 3:**
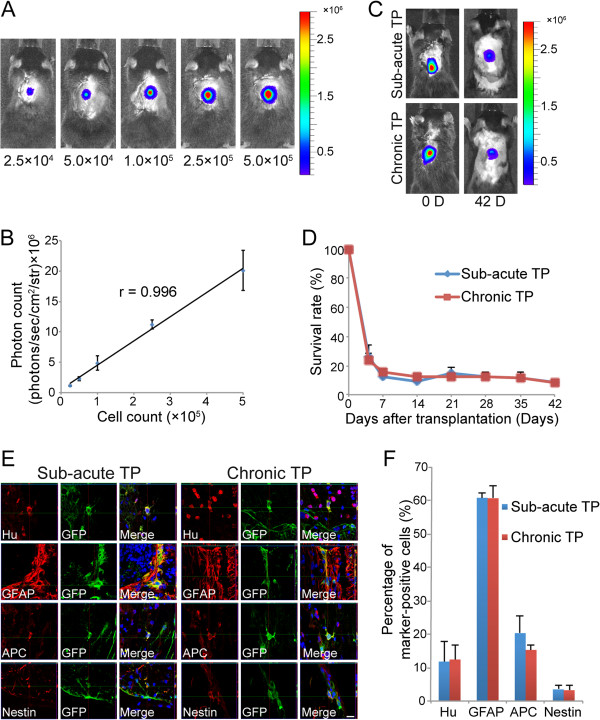
**Survival rate and differentiation phenotype of grafted cells in the sub-acute and chronic TP groups. A**, Representative *in vivo* BLI of naïve mice transplanted with various numbers of NS/PCs. **B**, The number of NS/PCs and the measured photon count were significantly correlated *in vivo*. Values are means ± SEM (n = 3). **C**, Representative *in vivo* BLI of the sub-acute and chronic TP groups immediately and 42 days after transplantation. **D**, Quantitative analysis using BLI revealed no significant difference in the survival rate of grafted cells between the sub-acute and chronic TP groups up to 42 days after transplantation. Values are means ± SEM (n = 10). **E**, GFP^+^ grafted cells differentiated into Hu^+^ neurons, GFAP^+^ astrocytes, and APC^+^ oligodendrocytes in the sub-acute and chronic TP groups. **F**, The differentiation rate of grafted cells into the three neural cell lineages did not significantly differ between the sub-acute and chronic TP groups 42 days after transplantation. Only a small number of nestin^+^ neural progenitor cells was detected in the sub-acute and chronic TP groups. Values are means ± SEM (n = 3). Scale bar: 10 μm in (C).

BLI analysis only detects luminescent photon signals from living cells, and the number of NS/PCs and the photon count *in vitro* were significantly correlated. To investigate whether similar correlativity was observed *in vivo*, various numbers of NS/PCs (approximate range 2.5 × 10^4^ to 5 × 10^5^ cells) were transplanted into the intact spinal cord of mice (Figure 3A). These data revealed that the photon count was significantly proportional to the number of grafted cells *in vivo* (Figure 3B).

We next analyzed the survival rate of the grafted cells on a weekly basis until 42 days after transplantation using BLI (Figure 3C). At 7 days after transplantation, BLI analysis revealed that the survival of grafted cells was reduced to 12.4 ± 5.6% in the sub-acute TP group and to almost the same level in the chronic TP group (15.2 ± 2.9%). At 42 days after transplantation, approximately 8% of the cells survived in both the sub-acute and chronic groups (8.6 ± 2.6% vs. 8.3 ± 1.9%). The survival rate of grafted cells did not significantly differ between the sub-acute and chronic TP groups at any time point examined (Figure 3D).

To evaluate the differentiation phenotype of the grafted cells *in vivo*, immunostaining for cell markers was performed 42 days after transplantation for both the sub-acute and chronic groups. Green fluorescent protein (GFP)^+^ grafted cells differentiated into all three neural lineages in both groups (Figure 3E). Quantitative analyses revealed that in the sub-acute and chronic TP groups, most of the grafted cells differentiated into GFAP^+^ astrocytes (60.8 ± 1.6% and 60.7 ± 3.7%), followed by adenomatous polyposis coli antigen (APC)^+^ oligodendrocytes (20.3 ± 5.1% and 15.3 ± 1.3%) and Hu^+^ neurons (11.8 ± 6.0% and 12.4 ± 4.2%). The differentiation rates of neurons, astrocytes, and oligodendrocytes did not significantly differ between the sub-acute and chronic TP groups (Figure 3F). Nestin-positive cells represented around 3% of the grafted cells in both TP groups.

### Grafted cells were limited to the lesion epicenter in the chronic TP group due to extensive glial scar formation

**Figure 4:**
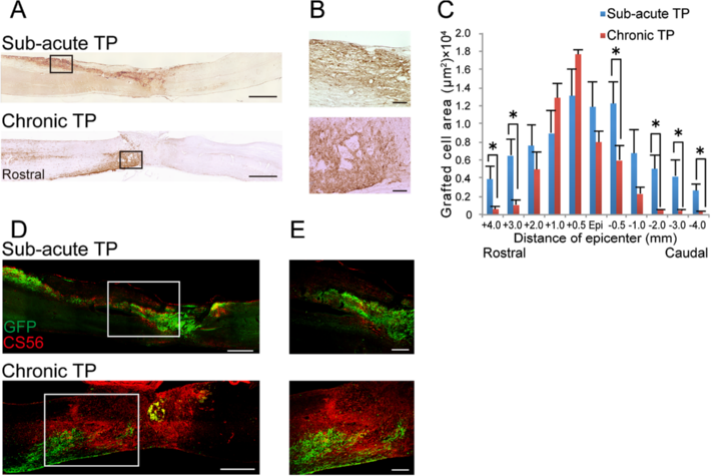
**Distribution of grafted cells in the sub-acute and chronic TP groups. A**, Representative images of GFP-immunostained sagittal sections in the sub-acute and chronic TP groups 42 days after transplantation. **B**, Higher-magnification images of the boxed areas in (**A**). **C**, Quantitative analysis of the GFP^+^ grafted cell area in axial sections 42 days after transplantation. Grafted cells were located at the epicenter, rostral, and caudal sites in the sub-acute TP group, whereas they were limited to the lesion epicenter in the chronic TP group. Values are means ± SEM (n = 4). ^*^P < 0.05. **D**, Representative images of GFP- and CS56-immunostained sagittal sections from the sub-acute and chronic TP groups. **E**, Higher-magnification images of the boxed area in (**D**). In the sub-acute TP group, the GFP^+^ grafted cells migrated away from the graft site due to less accumulation of CS56^+^ CSPG around the lesion site, while the robust CSPGs prevented further migration of the grafted cells in the chronic TP group. Scale bar: 1000 μm in (**A**), 100 μm in (**B**), 500 μm in (**D**) and 200 μm in (**E**).

To examine whether the prominent glial scarring seen in the chronic TP group affected the distribution of grafted cells, we performed immunostaining for GFP in both TP groups. In sagittal sections of the sub-acute TP group, GFP^+^ grafted cells were found at the epicenter and at rostral and caudal sites, whereas in the chronic TP group, grafted cells were seen almost exclusively at the lesion epicenter (Figure 4A, B). Quantitative analysis of the GFP^+^ areas in the axial sections revealed that the GFP^+^ area in the sub-acute TP group, which spread from 3 mm rostral to 2 mm caudal from the lesion epicenter, was significantly larger than that in the chronic TP group (Figure 4C). Moreover, to clarify what causes the difference of distribution of grafted cells, double immunostaining for GFP and CS56 was performed in both TP groups. The GFP^+^ grafted cells were surrounded and enclosed by CS56^+^ CSPG areas at the lesion site in the chronic TP group, whereas the grafted cells distributed beyond CSPGs areas due to less accumulation of CSPG around the lesion site in the sub-acute TP group (Figure 4D, E).

### NS/PC transplantation at the sub-acute phase, but not at the chronic phase, contributes to the preservation and/or enhancement of myelination and axonal growth

**Figure 5:**
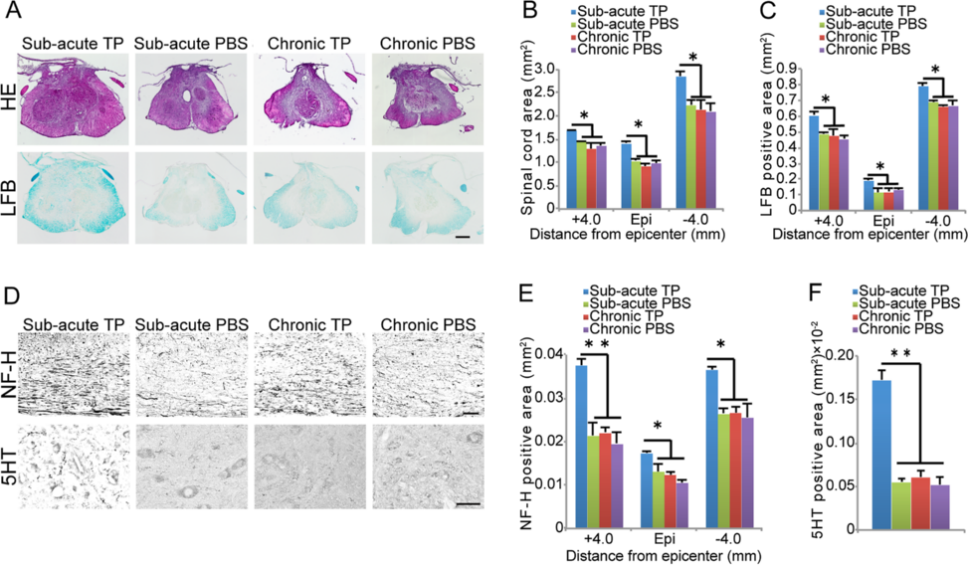
**Comparison of the effects of NS/PC transplantation between the sub-acute and chronic TP groups. A**, Representative HE- and LFB-stained images of axial sections at the lesion epicenter in the sub-acute TP, chronic TP, and both control groups. **B**, Significant atrophy of the spinal cords was observed in the chronic TP and control groups compared to that of the sub-acute TP group. Values are means ± SEM (n = 3). ^*^P < 0.05. **C**, The sub-acute TP group demonstrated a significantly larger myelinated area than the chronic TP and control groups. Values are means ± SEM (n = 3). ^*^P < 0.05. **D**, Representative images of sagittal sections stained for NF-H at the rim of the lesion epicenter and of axial sections stained for 5HT at the lumbar intumescence in the sub-acute TP, chronic TP, and each control group. **E**, Greater area of NF-H^+^ neuronal fibers was observed in the sub-acute TP group than in the chronic TP group or control groups. Values are means ± SEM (n = 3). ^*^P < 0.05, ^**^P < 0.01. **F**, Significantly larger areas of 5HT^+^ serotonergic fibers were detected in the sub-acute TP group than in the chronic TP or control groups. Values are means ± SEM (n = 3). ^**^P < 0.01. Scale bar: 100 μm in (**A**) and (**D**).

To compare the effects of NS/PC transplantation on the injured spinal cord between the sub-acute and chronic groups, axial sections were examined histologically using hematoxylin-eosin (HE) staining (Figure 5A). In sections at the lesion epicenter and 4 mm rostral and caudal to it, the HE-stained images revealed significant atrophy of the spinal cord in the sub-acute phosphate-buffered saline (PBS) group compared to the sub-acute TP group. However, the experimental transverse area of the spinal cord did not significantly differ between the chronic TP and PBS groups (Figure 5B). In addition, the sub-acute TP group demonstrated a significantly larger myelinated area compared to the sub-acute PBS group at the lesion epicenter and in sections 4 mm rostral and caudal to the epicenter (Figure 5A), whereas the myelinated area did not significantly differ between the chronic TP and PBS groups (Figure 5C).

Next, to investigate axonal growth after NS/PC transplantation, immunostaining for 200 kDa neurofilament (NF-H) and 5-hydroxytryptamine (5HT) was performed in all experimental groups (Figure 5D). The NF-H^+^ areas at the lesion site as well 4 mm rostral and caudal to it significantly differed between the sub-acute TP group and the other groups (Figure 5E). Furthermore, the sub-acute TP group demonstrated a significantly larger area of 5HT^+^ serotonergic fibers in the distal cord compared to the other three groups (Figure 5F). By contrast, the chronic TP group demonstrated no significant differences in the NF-H^+^ and 5HT^+^ areas compared to the chronic PBS group.

### NS/PC transplantation at the sub-acute phase, but not at the chronic phase, promotes motor function and electrophysiological recovery after SCI

**Figure 6:**
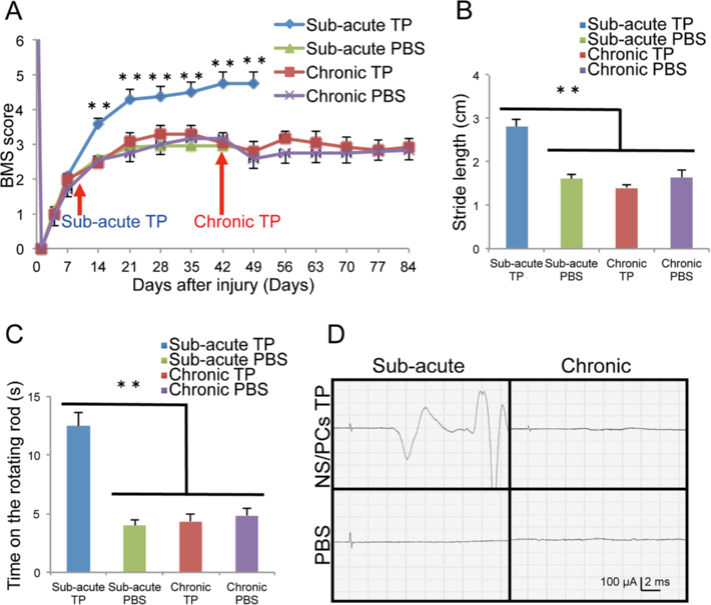
**Motor function and electrophysiological recovery after NS/PC transplantation. A**, Hindlimb motor function was assessed weekly by BMS score. The sub-acute TP group exhibited significantly better functional recovery than the vehicle control group at 14 DPI and thereafter, whereas no significant difference was observed between the chronic TP group and the vehicle control group. Values are means ± SEM (n = 10). ^**^P < 0.01. **B**, Treadmill gait analysis using the DigiGait system revealed a significantly longer stride in the sub-acute TP group than in the chronic TP group or either control group 42 days after cell transplantation or PBS injection. Values are means ± SEM (sub-acute TP n = 10, chronic TP and each control n = 8). ^**^P < 0.01. **C**, The sub-acute TP group walked for a significantly longer time on the rotating rod than the chronic TP group or either control group 42 days after cell transplantation or PBS injection. Values are means ± SEM (n = 10). ^**^P < 0.01. **D**, Representative profiles of MEP in the sub-acute TP, chronic TP, and both control groups 42 days after cell transplantation or PBS injection.

Finally, we evaluated locomotor functional recovery by Basso Mouse Scale (BMS) score, Rotarod testing, and DigiGait. We confirmed that the mice exhibited complete paralysis of the hindlimbs by a BMS score of 0 at 1 DPI. At 7 DPI, the hind limb locomotor functions had recovered spontaneously to an approximate BMS score of 2, and plateaued at an approximate BMS score of 3, in all experimental groups except the sub-acute TP group. In the sub-acute TP group, significantly greater functional recovery was observed compared to the sub-acute PBS group at 14 DPI and thereafter. By contrast, the chronic TP group did not show any functional recovery, with BMS scores comparable to those of the chronic PBS group (Figure 6A).

The gait performance of the mice in each group was analyzed using the DigiGait system at 42 days after cell transplantation or PBS injection. In both PBS control groups and the chronic TP group, a subset of experimental animals (2 of 10 mice in each group) were unable to walk sufficiently well on a treadmill moving at 7 cm/s to perform the test, but all the mice in the sub-acute TP group could perform the test. Gait analysis revealed a significantly longer stride length in the sub-acute TP group than in the sub-acute PBS group, whereas no significant difference in stride length was detected between the chronic TP and either PBS group (Figure 6B). Consistent with these findings, in the Rotarod test at 42 days after cell transplantation or PBS injection, the mice in the sub-acute TP group remained on the rotating rod for a significantly longer time than those of the sub-acute PBS group, but no significant difference was observed between the chronic TP group and either PBS group (Figure 6C).

Electrophysiological examinations performed at 42 days after cell transplantation or PBS injection by motor-evoked potential (MEP). MEP waves were detected in all mice of the sub-acute TP group, and the average signal-to-response latency was 6.1 ± 0.2 ms and the average first wave amplitude was 134.0 ± 31.5 mv. However no MEP waves were observed at all in 5 out of the 7 mice in the chronic TP group and at all mice in the sub-acute and chronic PBS groups (Figure 6D). Taking the results of all the motor functional analyses together, no functional recovery of the hindlimbs was observed in the chronic TP group.

## Discussion

Previous studies have indicated that NS/PC transplantation at the sub-acute phase has therapeutic potential for SCI [1-7,18]. At the chronic phase, however, NS/PC transplantation does not result in functional recovery. Many researchers have sought to improve motor function in chronic SCI by NS/PC transplantation. However, most studies obtained no significant functional recovery, and no consensus exists concerning the survival and/or fate of grafted cells in chronic-phase transplantation [9-12]. For example, some reports demonstrated that cells grafted at the chronic phase of SCI have a poor survival rate [9,12], while Cusimano et al. reported no significant difference in survival rate between NS/PC transplantation at the sub-acute versus the chronic phase [11]. However, these findings were based on histological examinations only. To clarify this issue, we performed sub-acute- and chronic-phase transplantations of NS/PCs derived from transgenic mice that ubiquitously express ffLuc-cp156 on a C57BL/6J background [14] to treat SCI in adult mice. These NS/PCs showed strong bioluminescent and fluorescent signals originating from ffLuc-cp156 [14], enabling the easy detection of surviving grafted cells in living animals noninvasively by BLI [4,13] and confirmation of integrated cells within the injured spinal cord by immunohistology for GFP without using a lentiviral vector.

Using cells from these mice, we demonstrated that the NS/PCs grafted during the chronic phase of SCI had a similar survival rate to those transplanted at the sub-acute phase. We also observed no significant difference between the two groups in the differentiation phenotypes of the grafted cells. The survival and/or differentiation potential of grafted cells can change remarkably due to microenvironmental changes in the injured spinal cord [19]. A previous study showed that grafted NS/PCs mainly differentiate into astrocytes after transplantation at the acute phase [4]. Inflammatory cytokines, such as IL-1β, IL-6, and TNF-α, also increase dramatically in the injured spinal cord during the acute phase [20,21], and these cytokines induce NS/PCs to differentiate into astrocytes. Moreover, growth factors affect the fate of grafted NS/PCs. For example, EGF, FGF, NT3, and PDGF have demonstrated beneficial effects on the survival of NS/PCs [10,12,22]. HGF and PDGF promote neuronal differentiation [23,24], whereas CNTF enhances glial differentiation *in vitro*[25]. To assess microenvironmental changes in the injured spinal cord, we performed a microarray analysis to exhaustively compare the gene expression profiles between the sub-acute and chronic phases of SCI. The expression levels of all cytokines and growth factors did not significantly differ, consistent with our BLI and histological results on the survival and/or fate of the grafted cells. These data suggested that the microenvironments of the injured spinal cord were not significantly different at the sub-acute and chronic SCI phases with respect to the expression of factors known to influence the survival rate and differentiation of grafted NS/PCs.

Many reports concern possible mechanisms underlying the therapeutic effects of NS/PC transplantation for SCI; for example, grafted cells contribute to the reconstruction of neural circuits and remyelination [2,6,17,26]. NS/PCs also secrete neurotrophic factors, which support the survival of host neural cells and enhance axonal growth and angiogenesis [27-30]. In this study, motor function recovery was observed in the sub-acute TP group, but not in the chronic TP group. Consistent with this result, more NF-H^+^ neuronal fibers and 5HT^+^ serotonergic fibers, which are involved in motor function recovery [30-32], were observed in the sub-acute TP group than in the sub-acute PBS group. Moreover, Luxol Fast Blue (LFB) staining revealed significantly larger myelinated areas in the sub-acute TP group than in the sub-acute PBS group. By contrast, no significant differences in NF-H^+^ neuronal fibers, 5HT^+^ serotonergic fibers, or LFB^+^ myelinated areas were observed between the chronic TP and chronic PBS groups. Furthermore, no MEP waves were detected in most mice in the chronic TP group.

As a possible reason for these findings, we refer to the poor migration of grafted cells in the chronic TP group. Since glial scar formation prevented grafted NS/PCs from migrating away from the graft site and integrating with the host spinal cord, the regeneration of neural circuits, remyelination, and/or preservation of host neural cells did not occur in areas rostral and caudal to the injured spinal cord of the chronic TP group. Consistent with this scenario, many previous reports indicate that insufficient migration of grafted cells results in poor functional recovery after SCI [33,34].

Our microarray and histological analyses suggested that more macrophages or microglial cells, including a substantial number of M2 macrophages, were distributed around the lesion epicenter at the sub-acute phase than at the chronic phase. In agreement with this finding, previous studies have reported that infiltration of macrophages and microglial cells peaks at approximately 7 DPI [35,36]. Moreover, M2 macrophages have an anti-inflammatory role and promote axonal growth after SCI [37-39], and their phagocytic activity was shown to contribute to tissue repair in the injured CNS by reducing myelin debris [40-42]. In addition, Busch et al. demonstrated that activated macrophages induced axonal dieback in the injured CNS [43], which was prevented by switching the infiltrating macrophages from M1 to M2 phenotype [39]. Furthermore, grafted NS/PCs also exert immunomodulatory effects through interactions with infiltrating immune cells [44,45]. Especially in the sub-acute phase, grafted NS/PCs can affect the properties of infiltrating macrophages, inducing their shift from M1 to M2 in the injured spinal cord [11,39]. Therefore, NS/PCs transplanted at the sub-acute stage may alter the microenvironment to favor M2, resulting in a synergistic effect that supports the increase in neuronal fibers and functional recovery. These findings indicate that a novel immunomodulatory strategy, such as the administration of a mediator of phenotypic switching from M1 to M2 or the combined transplantation of NS/PCs and M2 macrophages, may have therapeutic potential in chronic SCI. In contrast, previous studies reported that M1 macrophages impairs recovery of SCI through the production of oxidative metabolites or pro-inflammatory cytokines including TNF-α, which induced the apoptotic cell death of neural precursor cells [42,46,47]. However, the expressions of genes associated with M1 macrophages showed no significant difference among the sub-acute and chronic phases. These findings are consistent with the similar survival rate of the grafted NS/PCs in both TP groups.

Taken together, these results suggest that during the chronic phase of SCI, the injured spinal cord microenvironment appears to be unfavorable for the therapeutic mechanisms of NS/PC transplantation, owing to extensive glial scarring [48] and/or the phenotype of infiltrating macrophages. Accordingly, to improve the therapeutic potential of NS/PC transplantation performed at the chronic phase, altering the microenvironment in the injured spinal cord is likely to be important. For example, suppression of axonal growth inhibitors may improve the microenvironment. CSPGs, which are the most prevalent axonal growth inhibitors, are produced by reactive astrocytes and involved in glial scarring [48]. Chondroitinase ABC (ChABC), which digests CSPG, promotes axonal growth and the migration of grafted NS/PCs [49,50]. Karimi-Abdolrezaee et al. described that CSPGs hinder the survival of grafted cells and the combined therapy of ChABC administration and NS/PCs transplantation promotes the migration and integration of grafted cells [51]. While our data demonstrated that CSPGs prevent the migration of grafted cells, but show no influence on their survival, we have great hopes that a combination of NS/PC transplantation and the administration of a suppressor of axonal growth inhibitor may be effective for inducing functional recovery in chronic SCI.

## Conclusions

Microenvironmental changes in SCI affect the survival and/or fate of NS/PCs grafted into the injured spinal cord. The survival rate and differentiation phenotype of grafted cells were similar between chronic-phase and sub-acute-phase transplantation, with no significant difference in the secretion of cytokines and growth factors in the host environment between the two phases. However, the distribution of grafted cells was restricted by the robust glial scar that formed around the lesion epicenter at the chronic phase. Consequently, no functional recovery was observed in this phase. Additionally, in the sub-acute phase, M2 macrophages, which infiltrated predominantly in this phase, might contribute to the functional repair upon NS/PC transplantation. Alteration of the microenvironment in SCI, focusing on the glial scar and the inflammatory phenotype, appears to be the best possible solution to maximize the therapeutic potential of NS/PC transplantation in the chronic phase.

## Methods

### NS/PC culture and analysis

NS/PCs were cultured and expanded, as previously reported [52]. Briefly, the striata of transgenic mice ubiquitously expressing ffLuc-cp156, a fusion protein of firefly luciferase and a circularly permuted Venus protein [14], were dissociated using a fire-polished glass pipette on embryonic day 14. Venus is a fluorescent protein with fast and efficient maturation that was originally engineered from GFP [53], and therefore grafted cells can be detected as fluorescent Venus signals using anti-GFP antibody [17,26]. Dissociated cells were collected by centrifugation and re-suspended in culture medium, which consisted of Dulbecco’s modified Eagle medium/F12 (Sigma-Aldrich, St. Louis, MO, USA) supplemented with a previously described hormone mixture [52]. Human recombinant FGF-2 (Peprotech, Rocky Hill, NJ, USA) and EGF (Peprotech) (20 ng/ml each) were added every 2 days. The cells formed floating cell clusters (neurospheres) within 2–3 days. After propagation for 3 passages, the neurospheres were used for *in vitro* BLI, differentiation, and proliferation assays, and for cell transplantation.

For differentiation analysis, the neurospheres were plated onto poly-L-ornithine/fibronectin-coated 8-well chamber slides (Iwaki; Asahi Glass Co Ltd., Tokyo, Japan) at a density of 1 × 10^5^ cells/ml and cultured in culture medium without serum or growth factors at 37°C in 5% CO_2_ and 95% air for 7 days. The differentiated cells were then fixed with 4% paraformaldehyde in 0.1 M PBS and stained with the following primary antibodies for immunocytochemistry: anti-Tuj-1 (mouse IgG, 1:1000, Sigma-Aldrich), anti-GFAP (rat IgG, 1:1000, Invitrogen, Carlsbad, CA, USA), and anti-CNPase (mouse IgG1, 1:1000, Sigma-Aldrich). Nuclei were stained with Hoechst 33258 (10 μg/ml, Sigma-Aldrich). *In vitro* images were obtained using a fluorescence microscope (BZ-9000; Keyence Co., Osaka, Japan).

The proliferation assay was performed by measuring ATP, which indirectly reflects the number of viable cells [15,17,54]. In brief, NS/PCs were first incubated in culture medium without serum or growth factors in 48-well cell-culture plates (Corning Inc., Corning, NY, USA) at 37°C in 5% CO_2_ and 95% air for 24 or 72 h. D-luciferin was then added to each well, and the luminescent signal was detected immediately using a Xenogen-IVIS spectrum cooled charged-coupled device (CCD) optical macroscopic imaging system (Caliper Life Sciences, Hopkinton, MA, USA). To determine the population doubling time, the ATP assay was modified, as described elsewhere [15,17,54].

### SCI model

Adult female C57BL/6J mice (8–10 weeks old, 18–22 g, n = 52; Clea, Tokyo, Japan) were anesthetized with an intraperitoneal (i.p.) injection of ketamine (100 mg/kg) and xylazine (10 mg/kg). After laminectomy at the 10th thoracic spinal vertebra (Th10), the dorsal surface of the dura mater was exposed. Contusive SCI was induced using a commercially available SCI device (IH Impactor, Precision Systems and Instrumentation, Lexington, KY, USA), as previously described [4]. This device creates a reliable contusion injury by rapidly applying a force-defined impact (60 kdyn) with a stainless steel-tipped impactor. All experiments were approved by the ethics committee of Keio University and fully in accordance with the Guide for the Care and Use of Laboratory Animals (National Institutes of Health, Bethesda, MD, USA).

### Microarray analysis

The injured mice were anesthetized and transcardially perfused with heparinized saline (5 U/ml) at 9 DPI or 42 DPI (n = 3 each). Dissected segments of spinal cord at the Th10 level were rapidly frozen and placed in TRIzol (Invitrogen). Total RNA was isolated using an RNeasy Mini Kit (Qiagen Inc., Hilgen, Germany), in accordance with the manufacturer’s instructions. As a control, samples of naïve spinal cord were harvested by the same protocol. For microarray analysis, RNA quality was assessed using a 2100 Bioanalyzer (Agilent Technologies Inc., Santa Clara, CA, USA), and 100 ng of total RNA was reverse transcribed, biotin labeled, and hybridized to a GeneChip® Mouse Genome 430 2.0 Array (Affymetrix Inc., Santa Clara, CA, USA). The array was then washed and stained in a Fluidics Station 450, according to the manufacturer’s instructions [55,56]. The microarrays were scanned using a GeneChip Scanner 3000 7G, and the raw image files were converted to normalized signal intensity values using the MAS 5.0 algorithm. PCA was carried out with Gene Spring GX software (Agilent Technologies Inc.), using the full set of normalized data. For the clustering analysis, the normalized data were narrowed down by the cut-off values of expression levels (>50) and by fold change (>1.5, versus the signal of intact spinal cord), and statistical analysis was performed using one-way ANOVA followed by the Tukey-Kramer test (*P* < 0.05). The heat map was visualized with Gene Spring GX.

### NS/PC transplantation

Various numbers of NS/PCs (approximate range 2.5 × 10^4^ to 5 × 10^5^ cells/2 μl) were transplanted into uninjured naïve mice (n = 3, each), and NS/PCs (5 × 10^5^ cells/2 μl) were transplanted at 9 DPI (sub-acute TP group, n = 10) or 42 DPI (chronic TP group, n = 10) as previously reported [4,16,17,26,30,57]. The NS/PCs were transplanted into the lesion epicenter with a glass micropipette at a rate of 1 μl/min using a Hamilton syringe (25 μl) and a stereotaxic microinjector (KDS 310, Muromachikikai Co. Ltd., Tokyo, Japan). PBS (2 μl) was similarly injected into the lesion epicenter of the control mice at each time point (sub-acute and chronic PBS groups, n = 10 each).

### Bioluminescence imaging

A Xenogen-IVIS spectrum CCD optical macroscopic imaging system was used for *in vitro* and *in vivo* BLI as previously reported [4,16,17]. In brief, the signal intensity of NS/PCs *in vitro* was assessed using plated cells at various cell numbers (approximate range 1 × 10^2^ to 1 × 10^4^ cells/well), and BLI was performed immediately after adding D-luciferin (150 μg/ml) (n = 3). The integration time was fixed at 1 min for each image.

*In vivo* imaging was performed 15 min after the i.p. injection of D-luciferin (0.3 mg/g body weight) with the field-of-view set at 13.2 cm, because the photon count was most stable during this period. The intensity peaked between 10 and 30 min after the i.p. injection of D-luciferin. The integration time was fixed at 5 min for each image. All images were analyzed with Living Image software (Caliper Life Sciences), and the optical signal intensity was expressed as photon flux (photon count) in units of photons/s/cm^2^/steradian. Each result was displayed as a pseudo-colored photon count image superimposed on a gray-scale anatomic image. To quantify the measured light, a region of interest was defined in the cell-implanted area, and all values at the same region of interest were examined.

### Behavioral analyses

The motor function of each mouse was evaluated weekly using the BMS up to 49 DPI in the sub-acute TP and PBS groups and up to 84 DPI in the chronic TP and PBS groups (n = 10 per group) [58]. This assessment was performed by two investigators blinded to the identity of the experimental mice.

Motor coordination was evaluated using a rotating rod apparatus (Rotarod, Muromachikikai Co., Ltd.), which consisted of a plastic rod (3 cm diameter, 8 cm length) with a gritted surface flanked by two large discs (40 cm diameter) (n = 10 per group). At 42 days after cell transplantation or PBS injection, each mouse was placed on the rod while it rotated at 20 rpm for 2 min sessions [59]. Three trials were conducted, and the maximum number of seconds the mouse stayed on the rod was recorded.

Gait analysis was performed using the DigiGait system (Mouse Specifics, Quincy, MA, USA) (n = 10 per group) [17,26,60]. Each mouse demonstrated weight-supported hindlimb stepping at 42 days after cell transplantation or PBS injection. The stride length was determined on a treadmill set to a speed of 7 cm/s.

### Electrophysiology

Electrophysiological experiments were performed using a Neuropack S1 MEB-9402 (Nihon Kohden, Tokyo, Japan) at 42 days after cell transplantation or PBS injection (n = 7 per group) [26]. The animals were anesthetized with an i.p. injection of ketamine (60 mg/kg) and xylazine (6 mg/kg), and the stimulation was applied through the occipitocervical area of the spinal cord and the hindlimb by needle electrodes. The active electrode was placed in the quadriceps muscle belly, and the reference electrode was placed near the distal quadriceps tendon. The ground electrode was placed on the tail. A stimulus of 0.4 mA intensity, 0.2 ms duration, and 1 Hz interstimulus interval were used. The latency was measured as the length of time from the stimulus to the onset of first response wave. The amplitude was measured from the initiation point of the first response wave to its highest point.

### Histological analyses

Injured animals were deeply anesthetized and transcardially perfused with 4% PFA in 0.1 M PBS at 9 DPI or 42 DPI (n = 3 each). The treated animals were similarly prepared 42 days after cell transplantation or PBS injection. The spinal cords were removed, postfixed overnight in 4% PFA, soaked overnight in 10% sucrose, followed by 30% sucrose, embedded in Optimal Cutting Temperature compound (Sakura Finetechnical Co., Ltd., Tokyo, Japan), frozen, and sectioned in the sagittal or axial plane at 12 μm thickness on a cryostat (CM3050 S; Leica Microsystems, Wetzlar, Germany). The injured spinal cord sections were histologically evaluated by HE staining and immunohistochemistry, followed by quantitative analysis. The sections of transplanted spinal cord were subjected to HE staining, LFB staining, and immunohistochemistry followed by quantitative analyses.

For immunohistochemistry, tissue sections were stained with the following primary antibodies: anti-GFP (rabbit IgG, 1:200; Frontier Institute, Hokkaido, Japan), anti-Hu (human IgG, 1:1000, a gift from Dr. Robert Darnell, The Rockefeller University, New York, NY, USA), anti-GFAP (rat IgG, 1:200, Invitrogen), anti-APC CC-1 (mouse IgG, 1:200; Calbiochem, San Diego, CA, USA), anti-nestin (mouse IgG1, 1:500; BD Bioscience Pharmingen, San Jose, California, USA), anti-CS56 (a marker for CSPG, mouse IgM, 1:200; Sigma-Aldrich), anti-Iba1 (a marker for microglia/macrophages, rabbit IgG, 1:200; Wako Pure Chemical Industries, Osaka, Japan), anti-arginase-1 (a marker for M2 macrophages, goat IgG, 1:200; Santa Cruz Biotechnology, Santa Cruz, CA, USA), anti-LAMP2 (a marker for endosomes or lysosomes, rat IgG, 1:200; Abcam, Cambridge, UK), anti-NF-H (mouse IgG1, 1:200; Chemicon, Millipore, Billerica, MA, USA), and anti-5HT (goat IgG, 1:200; Immunostar, Hudson, WI, USA). For immunohistochemistry with anti-GFP, -NF-H, and -5HT, a biotinylated secondary antibody (Jackson Immunoresearch Laboratory Inc., West Grove, PA, USA) was used after exposing the sections to 0.3% H_2_O_2_ for 30 min at room temperature to inactivate endogenous peroxidases. The signals were enhanced with the Vectastain ABC kit (Vector Laboratories, Inc., Burlingame, CA, USA). Nuclei were stained with Hoechst 33258 (10 μg/ml, Sigma-Aldrich). All images were obtained using a fluorescence microscope (BZ 9000; Keyence Co.) or a confocal laser scanning microscope (LSM 700; Carl Zeiss, Munich, Germany).

### Quantitative analyses

Quantitative analyses of the histological findings (HE, LFB staining, and immunostaining for CS56, Iba1, NF-H, 5HT, GFP/each phenotypic marker, and arginase-1/Iba1) were performed using a BZ 9000 microscope and Dynamic Cell Count BZ-HIC software (Keyence Co.). The threshold values were maintained at a constant level for all analyses. The GFP^+^ area was quantified using images of axial sections of the lesion epicenter and 0.5 mm, 1.0 mm, 2.0 mm, 3.0 mm, and 4.0 mm rostral and caudal to the epicenter at 100× magnification (n = 4 each). To determine the spinal cord area, HE-stained images of axial sections of the lesion epicenter and 4.0 mm rostral and caudal to the epicenter at 100× magnification were used (n = 3 each). Quantitative analysis of the LFB^+^ area was similarly performed using axial sections of the lesion epicenter and 4.0 mm rostral and caudal to the epicenter at 100× magnification (n = 3 each). CS56^+^ areas as well as Iba1^+^ areas were quantified in the midsagittal sections of injured spinal cords at 100× magnification (n = 3 each).

To quantify NF-H^+^ fibers, four regions were automatically captured within the midsagittal sections of the lesion epicenter and 4.0 mm rostral and caudal to the epicenter at 400× magnification (n = 3 each). To assess the 5HT^+^ fibers, five automatically captured regions within the axial sections were analyzed at the lumbar intumescence, which was 6–8 mm caudal to the lesion epicenter (n = 3 each).

To quantify the proportion of each cell phenotype among the grafted cells *in vivo*, five regions were captured within sagittal sections at 200× magnification using an LSM 700 confocal laser scanning microscope. GFP and phenotypic marker double-positive cells were counted in each section (n = 3 each). To quantify the infiltrated M2 macrophages in the injured spinal cord, the number of Iba1 and arginase-1 double-positive cells was counted within five regions of sagittal sections of the lesion epicenter at 200× magnification with an LSM 700 confocal laser scanning microscope (n = 3 each).

### Statistical analysis

All data are reported as the mean ± SEM. An unpaired two-tailed Student’s *t* test was used to evaluate the differences between groups with respect to microarray gene expression profile, *in vivo* BLI analysis, *in vivo* differentiation assays, and analyses of the CS56^+^, Iba1^+^ and GFP^+^ areas, and arginase-1^+^/Iba1^+^ cells. One-way ANOVA followed by the Tukey-Kramer test for multiple comparisons was used in the analyses of the HE, LFB, NF-H^+^, and 5HT^+^ areas and the Rotarod and DigiGait results. Repeated-measures two-way ANOVA followed by the Tukey-Kramer test was used for the BMS analysis. *P* values < 0.05 were considered statistically significant.

## Abbreviations

NS/PCs: Neural stem/progenitor cells; SCI: Spinal cord injury; DPI: Days post-injury; BLI: Bioluminescence imaging; TP group: Transplanted group; ffLuc: Fluorescent protein-fused luciferase; Tuj-1: βIII tubulin; GFAP: Glial fibrillary acidic protein; CNPase: 2’3’-cyclic nucleotide 3’-phosphodiesterase; CSPG: Chondroitin sulfate proteoglycan; PCA: Principal component analysis; iNOS: Inducible nitric oxide synthase; APC: Adenomatous polyposis coli antigen; HE: Hematoxylin-eosin; PBS: Phosphate-buffered saline; NF-H: 200 kDa neurofilament; 5HT: 5-hydroxytryptamine; BMS: Basso Mouse Scale; MEP: Motor-evoked potential; LFB: Luxol Fast Blue; ChABC: Chondroitinase ABC.

## Competing interests

The authors have declared that no competing interests exist.

## Authors’ contributions

SN, AY, OT, HO, and MN designed the research; SN, AY, HI, MT, and HE performed research; SN, AY, and HI analyzed the data; SN, HO, and MN wrote the paper; and MN and HO supervised all the experiments. All authors read and approved the final manucript.
